# Integrating a Web-Based Self-Management Tool (Managing Joint Pain on the Web and Through Resources) for People With Osteoarthritis-Related Joint Pain With a Web-Based Social Network Support Tool (Generating Engagement in Network Involvement): Design, Development, and Early Evaluation

**DOI:** 10.2196/18565

**Published:** 2020-11-26

**Authors:** Paul Clarkson, Ivaylo Vassilev, Anne Rogers, Charlotte Brooks, Nicky Wilson, Jem Lawson, Jo Adams

**Affiliations:** 1 NIHR ARC Wessex Centre for Sport, Exercise and Osteoarthritis Research Versus Arthritis School of Health Sciences, University of Southampton Southampton United Kingdom; 2 Social Networks Health and Wellbeing Research Group School of Health Sciences, University of Southampton Southampton United Kingdom; 3 School of Health Sciences University of Southampton Southampton United Kingdom; 4 King’s College Hospital NHS Foundation Trust London United Kingdom; 5 Centre for Sport, Exercise and Osteoarthritis Research Versus Arthritis Southampton United Kingdom

**Keywords:** joint pain, osteoarthritis, internet, self-management, social networks

## Abstract

**Background:**

Joint pain caused by osteoarthritis (OA) is highly prevalent and can be extremely debilitating. Programs to support self-management of joint pain can be effective; however, most programs are designed to build self-efficacy and rarely engage social networks. Digital interventions are considered acceptable by people with joint pain. However, many existing resources are not accessible for or developed alongside people with lower health literacy, which disproportionately affects people with OA.

**Objective:**

This study aims to design and develop an accessible digital self-management tool for people with joint pain and integrate this with an existing social network activation tool (Generating Engagement in Network Involvement [GENIE]) and to explore the feasibility of these linked tools for supporting the management of joint pain.

**Methods:**

The study was conducted in 2 phases: a design and development stage and a small-scale evaluation. The first phase followed the person-based approach to establish guiding principles for the development of a new site (Managing joint Pain On the Web and through Resources [EMPOWER]) and its integration with GENIE. People with joint pain were recruited from libraries, a community café, and an exercise scheme to take part in 3 focus groups. EMPOWER was tested and refined using think-aloud interviews (n=6). In the second phase, participants were recruited through the web via libraries to participate in a small-scale evaluation using the LifeGuide platform to record use over a 1-month period. Participants (n=6) were asked to complete evaluation questionnaires on their experiences. The NASSS (nonadoption, abandonment, scale-up, spread, and sustainability) framework was used to explore the feasibility of the sites.

**Results:**

The focus groups established guiding principles for the development of the tool. These included ensuring accessibility and relevance for people with OA-related joint pain and recognizing that joint pain is the reason for seeking support, trust, social facilitation, and goal setting. Think-aloud interviews identified issues with user experience and site navigation and the need for professional input for referral and goal setting, confusion, and tensions over the role of GENIE and site connectivity. Participants expected the sites to be specific to their pain-related needs. EMPOWER was accessed 18 times; 6 users registered with the site during the evaluation study. Participants mostly explored information pages on being active and being a healthy weight. Only one participant undertook goal setting and 4 participants visited the GENIE website.

**Conclusions:**

Using the NASSS framework, we identified the complexity associated with integrating EMPOWER and GENIE. The value proposition domain highlighted the technical and conceptual complexity associated with integrating approaches. Although identified as theoretically achievable, the integration of differing propositions may have caused cognitive and practical burdens for users. Nevertheless, we believe that both approaches have a distinct role in the self-management of joint pain.

## Introduction

### Background

Joint pain secondary to osteoarthritis (OA) causes disability for many people and can be associated with a loss of independence [1,2]. In the United Kingdom, a 7-year consultation (2004-2010) determined a prevalence of 8.75 million people with OA aged above 45 years [3]. The prevalence of OA increases with age, with substantial associated human and economic costs [4]. Given the projected increase in older adults across the European Union by 2080 [5], this impact is expected to grow. OA disproportionately affects lower socioeconomic groups [6]. Such groups have been found to have low levels of health literacy, which is associated with poorer health outcomes [7].

Self-management is defined as both the action of a person to actively engage with their own health treatment and a program for delivering health-promoting information to people with chronic conditions [8,9]. Active engagement with self-management is an essential part of everyday life for people living with a long-term condition (LTC) [9], such as OA. Therefore, it is relevant to understand how support for self-management could be optimized. Programs or interventions to support the daily management of LTCs can be effective. Small improvements in symptom control, including pain, have been reported in people with OA following self-management programs, although these effects may not translate into improved quality of life [10]. Barriers to effective OA self-management programs, reported by patients in primary care [11], include a lack of information from health care professionals, beliefs that OA cannot be improved, and negative perceptions about program formats. A key recommendation in most self-management programs for OA is to increase physical activity [12]. Although both face-to-face and digital interventions can effectively support the promotion of physical activity [13,14], digital interventions are accessible to a broader range of people [15] and acceptable as a method for supporting the self-management of joint pain [16]. Furthermore, digital tools for managing chronic conditions have been found to increase awareness and build capacity for people to better manage their condition [17].

Self-management interventions often combine multiple interacting components to improve health and well-being and commonly include behavioral change approaches [18]. Interventions that are designed using behavior change theory are considered effective at improving outcomes for people [19,20]. However, using this approach alone focuses only on an individual’s motivation to self-manage. Other approaches that use a social network approach, which seeks to improve engagement with existing network members and resources and build new connections in the community to meet the needs of individuals, also enhance self-management [18,21,22]. However, to date, these have not been included in OA self-management programs. A relational approach to self-management, focused on the interdependence between individuals and network-level processes, can assist in changing behavior, managing day-to-day practicalities, and sharing experiences [22]. Consequently, there is considerable potential for this approach to improve the effectiveness of self-management programs for people living with OA. An example of a facilitated network-centered approach is provided by a web-based tool called GENIE (Generating Engagement in Network Involvement) [23]. GENIE is an evidence-based intervention that aims to reconstruct existing relationships and build new connections through valued activities to develop a diverse social network. GENIE is most effectively delivered through a one-to-one interaction by a trained facilitator and includes 4 distinct stages [22,23]: (1) mapping an individual’s social network using concentric circles, (2) exploring activity and support preferences, (3) linking network members to their preferences, and (4) providing access to information on local resources linked to an individual’s preferences.

Deductive approaches to developing health care interventions are commonly used [24,25]. However, it is important to explore the needs of people living with the condition to ensure that the intervention is effective and acceptable to those who will ultimately use it [26]. One approach to achieve this is the *person-based approach* (PBA) [27].

### Aims and Objectives

This study aims to describe the development of a new web-based self-management intervention using the LifeGuide software, developed at the University of Southampton, which integrates a traditional evidence-based approach to supporting self-management, such as My Joint Pain (MJP) [28] with social network support using GENIE. The objectives of this study are as follows: (1) to design and develop, alongside user-led groups, a digital, personalized self-management program accessible to people with lower health literacy and joint pain; (2) to link this digital, personalized self-management program with the social network GENIE tool; and (3) to conduct an early evaluation of the self-management program and its integration with GENIE for people with joint pain.

## Methods

### Study Design

**Figure 1:**
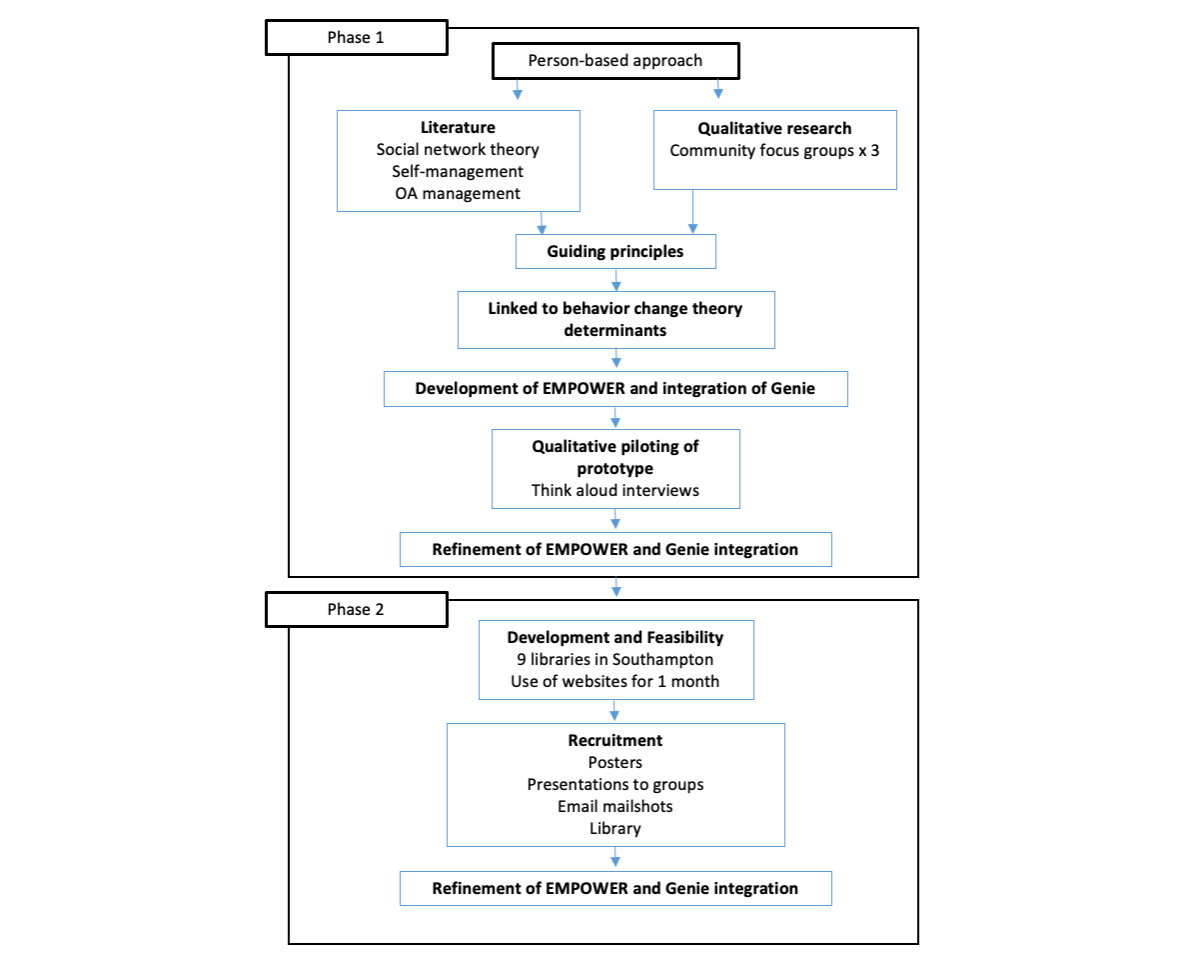
The person-based approach used for the design and development of the web-based intervention in phase 1 and small-scale evaluation using LifeGuide software. EMPOWER: Managing joint Pain On the Web and through Resources; Genie: Generating Engagement in Network Involvement; OA: osteoarthritis.

The study was divided into 2 phases: (1) design and development, including focus groups and think-aloud interviews and (2) a small-scale early evaluation of the intervention using LifeGuide software (Figure 1). Underpinning our design and development approach was the use of appropriate behavior change theory and the literature on social networks. These methods are discussed in the following sections.

### Ethics

Ethical approval to conduct the study was granted by the Faculty of Environmental and Life Sciences ethics committee at the University of Southampton (Ref. 40268.A1, 48286).

### The PBA

The PBA provides a systematic way to integrate theory with the lived experiences of people with LTCs to develop usable and engaging interventions [29]. In this project, the PBA was used to identify guiding principles for the development of the intervention by using and linking data collected from participants to relevant theories and existing research. Once prototypes were developed, qualitative approaches were used to further refine the intervention.

### Phase 1: Design and Development

To design and develop a new digital self-management tool, we invited people with joint pain to take part in focus groups to review and provide feedback on an existing web-based joint pain self-management tool for OA [28] alongside GENIE [30]. Participants were subsequently invited to participate in think-aloud interviews to review the newly created tool.

#### LifeGuide Software

The new digital self-management tool was developed using the LifeGuide software, which is an open-source software that enables the development of web-based resources without the need for programming experience. It was developed at the University of Southampton and has previously been used alongside the PBA for self-management interventions for asthma [31], hypertension [32], and weight loss [33].

#### MJP

MJP [28] is a web-based resource for people with OA-related joint pain. Developed by Arthritis Australia, it provides information to support the management of joint pain. The MJP asks participants to indicate the location of their pain on a visual body map and answer questions so that the content of the site can be tailored and made relevant to the individual. Participants can return to the site for follow-up visits to further individualize their management plan [28]. MJP was reviewed by the study participants, as it represented an up-to-date and novel web-based tool for people with OA [28]. However, the site requires a high level of health literacy, particularly in relation to the level and quantity of information provided [15,16]. Consequently, it was important to understand whether a more accessible site could be developed for UK-based users.

#### Recruitment

We recruited a convenience sample of people with joint pain from a variety of backgrounds and with different health literacy levels from community organizations in Southampton, United Kingdom. We used posters, presentations to groups, and drop-in sessions to support recruitment into the study. All participants were provided with a participant information sheet (PIS) that was designed to be accessible, with a reading level of <12 years [34]. A simplified and more visual information sheet also accompanied the full PIS. All participants signed a consent form before taking part and verbally consented before data collection. All study materials were reviewed by a patient and public involvement representative (JL) and a health literacy advisor (CB) before use. Participants were eligible to take part if they were aged >50 years and had self-reported joint pain. This age group was chosen because of the age-related increase in the prevalence of OA [4,35]. Self-reported joint pain reflects the fact that many people living in the United Kingdom with OA-related joint pain do not have a confirmed OA diagnosis [36].

#### Procedures

A total of 3 focus groups were conducted in community locations and at the University of Southampton in May and June 2018. Participants provided demographic information (age, gender, years of full-time education, highest level of education, and most recent occupation) and answered a question on health literacy (How confident are you at filling out medical forms by yourself? [37]). This health literacy question was found to be predictive of inadequate health literacy when compared with a larger validated measure—the Short Test of Functional Health Literacy in Adults [38]. Each focus group lasted 1 hour and was conducted by 2 members of the research team (a moderator [PC] and a note taker [CB or IV]) with experience of conducting focus groups. Participants were asked to review MJP and GENIE websites using their choice of a laptop or tablet. Immediately after viewing each site, participants were asked about their overall impressions. After viewing both sites, a further discussion, led by the moderator, was held using a topic guide. These focus group discussions provided information on the development of an initial version of the new digital self-management tool and its integration with GENIE.

Participants who had previously consented to participate in the think-aloud interviews were invited to review the initial version of the new tool. Think-aloud interviews were chosen to identify user responses to the content, style, and delivery of the new tool [27]. Before participating, each participant watched a short video demonstrating the think-aloud method. Interviews were conducted by one researcher (PC) at a time convenient to the user and were conducted either at the university or at the participant’s home. The interviews were conducted in January and February 2019 using a predesigned interview schedule of tasks related to the key aspects of the site. There was no previous relationship between the participants and the researchers.

Focus groups and think-aloud interviews were digitally recorded and transcribed verbatim. Transcripts were read repeatedly to deepen comprehension by 2 researchers (PC and IV), coded and categorized using content analysis [39] by PC, and discussed with IV, AR, and JA to establish guiding principles for intervention development and amendments within the constraints of the technology and time.

### Phase 2: Early Evaluation of the Integrated Tools

Following the modifications suggested by the interview participants, we conducted a small-scale early evaluation of Managing joint Pain On the Web and through Resources (EMPOWER) and its integration with GENIE. We used quantitative usability metrics, recorded in the LifeGuide platform and accessible to the research team, including the number and the time of visits to the site, time spent on each page and how a participant navigated through different pages, the use of various functions such as goal setting and email reminders, and text entered. We also aimed to establish the feasibility of the tools for use in a larger trial, based on the following criteria: acceptability, demand, implementation, practicality, integration, and limited efficacy [40]. A mixed methods approach is considered beneficial for developing digital interventions for people with lower levels of health literacy [41]. We aimed to recruit a diverse group of people from across the community through 9 local libraries in different city locations. However, no specific recruitment target was set, as we were interested in exploring recruitment feasibility for a future larger scale study. According to the English Indices of Deprivation, 5 recruitment locations were in the highest 2 deciles of deprivation. The others clustered around deciles 4, 5, and 6 [42].

We amended the age inclusion criteria to ≥18 years based on feedback during the development phase about being able to access such resources at an earlier age. EMPOWER was designed for people with OA; however, our development work indicated that many people will not have been given a formal diagnosis. We therefore used a symptomatic definition for recruitment, which was movement-related joint pain with morning stiffness that does not resolve within 30 minutes. Participants were also required to have access to an internet-enabled computer or tablet and be able to read English.

#### Recruitment

Recruitment was conducted using posters and library presentations and through an email newsletter to library users and city council staff. Potential participants had access to the study information sheet and instructions about how to find the EMPOWER site and take part. Participants were asked to confirm their eligibility and consent to participate in the study. Once registered, participants answered a series of demographic questions, including age, gender, OA diagnosis, and postcode (to explore deprivation indices). Participants were also asked to complete questionnaires on self-efficacy [43], health literacy [37,38], and the impact of joint pain symptoms [44].

#### Procedures

Participants had access to the full EMPOWER site for 1 month, including email reminders, external links, and an option to request a facilitated GENIE session. At the end of the month, registered participants received an email inviting them to return to EMPOWER to evaluate the content and feasibility of the tools, complete the health literacy question [37,38] and the impact of joint pain questionnaire [44], and attend an interview. EMPOWER was programmed to automatically show the evaluation questions when the participant signed into the site at the end of the month. Each registered participant received an e-gift voucher to thank them for participating. No changes were made to the interventions during the evaluation period.

To evaluate the complexity of the intervention, we drew on the first 4 domains of the NASSS (nonadoption, abandonment, scale-up, spread, and sustainability) framework [45]. This framework was developed through systematic reviews and case studies to identify appropriate domains, which were then tested with 10 programs across health and social care (Multimedia Appendix 1). The NASSS framework is considered particularly relevant for the evaluation of technological interventions [46]. Each domain can be classified as simple, complicated, or complex, with greater complexity representing a less chance for sustained adoption. Usability data were used to explore these domains.

## Results

### Phase 1: Design and Development—Focus Groups

In total, 11 people participated in the focus groups, which included groups of 5, 4, and 2. The participants were mostly women and aged 50 to 79 years. Just under half of the group reported that they had a secondary level of education (aged 12-16 years), whereas the others had a college (aged 16-18 years) or university education (>18 years). Most participants had higher health literacy levels (n=8; Table 1).

The results of the focus groups were divided into factors related to the usability of the sites and themes for intervention, development, and implementation. The following themes were identified: introductions or recommendations, flexibility, complementarity, tailoring, and important aspects of managing joint pain. Representative accounts are shown in Multimedia Appendix 2.

**Table 1:** Focus group and think-aloud interview participant demographics.

Participant	Age (years)	Gender	Years of full-time education	Highest educational level	Most recent occupation	Health literacy score^a^
P2^b^	70-79	Male	15	Secondary	Milkman	A little bit
P3	60-69	Female	10	Secondary	Nurse	Quite a bit
P5^b^	50-59	Female	12	College	Library supervisor	Quite a bit
P6^b^	60-69	Female	13	College	Craft tutor and child carer	Quite a bit
P7^b^	50-59	Female	19	University	Support worker	Extremely
P8	60-69	Female	12	College	Customer advisor	A little bit
P9	50-59	Male	12	Secondary	Road sweeper and toilet cleaner	A little bit
P10^b^	50-59	Male	11	Secondary	Information technology specialist	Quite a bit
P11	50-59	Male	6	College	Engineer	Extremely
P13^b^	50-59	Female	18	University	Community development officer	Extremely
P14	70-79	Female	12	Secondary	Receptionist and telephonist	Extremely

^a^Responses to health literacy question—extremely (likely high health literacy) to not at all (likely low health literacy).

^b^Participants took part in think-aloud interviews.

#### Introductions or Recommendations

Participants considered that contexts facilitating engagement with the websites could be associated with perceptions of trust in the site’s content; for example, a recommendation from a general practitioner (GP) would provide reassurance that the site was suitable. GENIE was also considered to have potential as a proactive tool when introduced by a social housing officer. The sites’ independence of commercial interests was important to participants.

#### Flexibility

Participants wanted self-management interventions to be sufficiently flexible to meet their needs at different times. Although participants reviewed the MJP website before the GENIE website during the focus groups, there were mixed opinions about the order in which the sites could be used. Some participants liked the idea of using GENIE with a facilitator first. Others found it difficult to initially see how it could help to manage their joint pain. For this reason, some participants felt that joint pain should be the starting point, with a site such as MJP, and lead onto GENIE.

#### Complementarity

The two websites (MJP and GENIE) were perceived as complementary to one another. Participants liked being able to find information on how to manage their joint pain while also having the opportunity to find people and resources that might help in their own community.

#### Tailoring

Participants were asked a series of questions on the MJP website to tailor the information on the site but found that the results fell short of their expectations in terms of personalization and relevance due to similar recommendations across the group. Participants disliked having to sign up immediately to perform this tailoring process, preferring to browse the site first. However, this process did reassure others that their painful joint was displayed on a visual body map.

#### Important Aspects of Managing Joint Pain

Participants considered web-based resources helpful in alleviating pressure on the National Health Service (NHS) but suggested that GPs would need to be aware of the sites. They highlighted the role of others in self-management and the need for relevant resources for friends and family. Furthermore, because of feelings of loneliness and isolation, it was deemed important to connect with others who experienced similar issues. Other important features for managing joint pain were discussed, including maintaining activity (partly for distraction from pain) and acquiring information about practical resources and support. Tracking personal progress and gaining emotional support to cope with pain were also considered important.

#### Identifying Guiding Principles for Design and Development of EMPOWER and Its Integration With GENIE

The guiding principles for the new self-management tool (EMPOWER) and its integration with GENIE were generated from focus group themes and research literature on the self-management of OA and are as follows:

- Accessible for people with different levels of health literacy.
- Relevant to users need at different times.
- Self-management support strategies beyond medical advice.
- Pain as a starting point.
- Being able to track progress.
- Trust in the resource.

These guiding principles were developed into design objectives for the project. Intervention features informed by behavior change theories were chosen to meet these objectives (Table 2).

**Table 2:** Design objectives, intervention features, and background literature or theory linked to the guiding principles.

Design objectives	Intervention features	BCT^a^ or previous literature
To ensure that the intervention is accessible for people with lower levels of health literacy	Ensure that the site and content are accessible and understandable Reduce complexity and jargon to enable the personal use of information Provide options to support users to apply relevant information and put advice into practice	Integrated model of health literacy [47]
To enable people with joint pain to gain advice and support that is relevant to them at different times	Integration of web and community resources to provide information and advice when it is required Links to community resources to connect web information with real-world application	Integrated theory of health behavior change [48]
To encourage people with joint pain to think about and engage with support in terms of their wider social network	Integration of the GENIE^b^ tool with the new joint pain self-management tool (EMPOWER^c^) Ensure that the benefits of social network support are highlighted	Integrated theory of health behavior change [48]
To develop an approach that recognizes joint pain as the rationale for seeking support	Ensure features and navigation through the sites that recognize joint pain as the motivation for accessing the intervention	Self-determination theory (extrinsic motivation—identified regulation) [49,50]
To encourage people to set goals to promote action and maintenance of self-management behaviors	Promote the creation of goals from information on the sites to develop behaviors for managing joint pain	Health action process approach [51] Goal setting theory [52,53]
To ensure that users consider the intervention to be trustworthy	Provide references for all information Provide information about the development of the intervention by people with joint pain, researchers, and health care professionals	No specific BCT but background on trust and reputational mechanisms [54,55]

^a^BCT: behavior change theory.

^b^GENIE: Generating Engagement in Network Involvement.

^c^EMPOWER: Managing joint Pain On the Web and through Resources.

#### Developing the Intervention

A new joint pain self-management intervention was created, called EMPOWER. To develop an initial prototype, we first explored the potential to integrate EMPOWER with GENIE. However, technical programming constraints prohibited this, and it was necessary to find another method for integration. In accordance with guiding principle 4, we established EMPOWER as the starting point for users, acting as a resource for managing joint pain. The aim of EMPOWER was to facilitate the adoption of and engagement with self-management behaviors relevant to an individual, through information and engagement with network support. This was emphasized on the EMPOWER home page through links to joint pain information in EMPOWER itself and information about the benefits of social network support and GENIE facilitation. EMPOWER was also designed to prompt users to think about how members of their networks might help with self-management throughout the site. This included space on each page to record information about people or activities that might help, which was automatically transferred to the goal setting pages for integration into personal plans (Multimedia Appendix 3). Each page also included a link to the GENIE database of activities and groups, enabling users to identify local resources that could be relevant for making the changes individuals identified as important. These pages opened in a separate browser to ensure that the sites could be used interchangeably.

The content of EMPOWER was informed by focus group discussions, research evidence or guidelines, and in collaboration with Arthritis Australia. All content was reviewed by expert clinicians working in musculoskeletal services and the research team, including clinicians, sociologists, and a health psychologist. Video content provided by an existing GENIE project [23], Arthritis Australia [28], and the Chartered Society of Physiotherapy, United Kingdom, was considered beneficial by participants. Access to in-depth information was provided through public links to trusted third-party sites, including Versus Arthritis [56] and the NHS [57], which opened in separate browser windows.

#### Think-Aloud Interviews

A total of 6 participants took part in the think-aloud interviews. Participant demographic information is shown in Table 1. Interviews lasted between 20 and 60 minutes, reflecting the diverse needs and interests of participants in relation to joint pain. The following themes were identified: user experience, professional involvement, understanding GENIE, tensions between EMPOWER and GENIE, and pain and goal setting. Participants highlighted particular issues associated with the technical and conceptual integration of EMPOWER and GENIE. This included confusion over whether any information entered on one site would influence the content of the other and a perceived conflict in approaches to self-management. Each theme is summarized below with illustrative accounts available in Multimedia Appendix 2.

#### User Experience

Participant feedback indicated that the overall navigation and integration of EMPOWER and GENIE needed improvement. Links to GENIE, which opened in separate windows, were confusing, particularly when participants were trying to return to EMPOWER. Most participants wanted EMPOWER to be more personalized and specific to their information needs. However, there were marked differences in opinion about the level of information provided. Differences were also reported about the site’s appearance, with one participant reflecting that the site aesthetic was too formal and another considering that this formality improved the look of the site.

#### Professional Involvement

Different views about health care professionals’ involvement in self-management and goal setting decisions were held. Some participants stated that it was important to draw on their own experience of managing joint pain, whereas others wanted a more prescribed approach. Furthermore, some participants were confused by perceived inconsistencies between some of the video content, which promoted the involvement of expert advice, and the wider self-management ethos of the sites.

#### Understanding of GENIE

Participants perceived that the role of GENIE was to locate groups and community content specifically linked to information within EMPOWER. However, although specific EMPOWER pages were linked to relevant GENIE pages, this was not sufficient to meet one participant’s expectations. Involving other network members was considered important, although inner resilience was also integral to management. Options to record how your network could help on the EMPOWER pages were confusing and not used by most participants, suggesting that engaging with social networks for self-management requires a more facilitated approach.

#### Tensions Between EMPOWER and GENIE

The link between EMPOWER and GENIE required more explanation, as participants were confused about whether the content from both sites was automatically transferred to the other for use. In particular, given the physical impact of joint pain, one participant was concerned that linking up with local groups and activities recommended through GENIE may not be relevant. Others found it difficult to see how network members could influence their pain.

#### Pain

Expectations of the sites were linked to participants’ joint pain needs. One participant expected more joint pain–specific content, whereas others wanted a greater focus on prevention to reduce the future impact of pain. Participants believed that given the varied nature of pain experiences, achieving relevance for all users would be difficult. Some of the participants did not recognize the term osteoarthritis.

#### Goal Setting

Goal setting was found to be potentially beneficial for some participants but only if linked with professional advice. One participant questioned the need for goal setting, whereas a further participant considered that the overwhelming amount of information made it difficult to select appropriate goals.

#### Refinement of EMPOWER and GENIE Integration

Amendments were made to EMPOWER and to its integration with GENIE following the think-aloud interviews. These included a new video on the EMPOWER home page, with explicit focus on improving the understanding of GENIE and its relevance within EMPOWER. User experience was also improved to ease tensions associated with navigating between EMPOWER and GENIE. Changes were made to the content and language used, such as replacing the term “network” with “people around you” (Multimedia Appendix 4) to improve users’ understanding of social network support. A greater focus on the lived experience of people with joint pain was achieved through the inclusion of links to videos from the Healthtalk website [58]. The reported usability issues were improved through greater signposting to guide user navigation.

### Phase 2: Early Evaluation of the Integrated Tools

The EMPOWER site was accessed 18 times for at least 10 seconds in the month it was available. Of the 17 eligible participants, 9 consented to participate in the study; however, only 6 registered and used the site’s functions. Overall, 2 participants returned to the site twice within the month of the initial registration.

#### Registered Users

Table 3 displays the registered users’ demographics. The participants were mostly female and aged 52 to 68 years. Overall, 3 participants reported a diagnosis of OA and indicated a greater impact of their symptoms on daily life. All participants reported joint pain in more than one joint, with 3 participants indicating pain in more than 5 joints. Most participants had a higher level of health literacy, whereas postcode data indicated that participants predominantly lived in areas with a lower level of deprivation (4/6, ≥5th decile [42]). Responses to the self-efficacy questionnaire showed a wide range of perceived self-efficacy (mean 5.5 [SD 2.1]; range 2.8-8.0).

**Table 3:** Registered participant demographics (phase 2).

Participant ID	Gender	Age	Osteoarthritis diagnosis	Impact of joint symptoms^a^	Health literacy^b^	Self-efficacy, mean (SD)^c^
P15	Female	54	No	Slightly	Quite a bit	7.7 (1.2)
P16	Female	66	No	Slightly	Extremelyc	8.0 (1.1)
P17	Male	68	Yes, physiotherapist	Moderately	Extremely	5.8 (1.9)
P18	Female	68	Yes, general practitioner	Severely	Quite a bit	3.3 (0.5)
P19	Female	68	No	Moderately	Extremely	5.3 (0.8)
P20	Female	52	Yes, doctor at surgery, scans, etc	Severely	Somewhat	2.8 (1.2)

^a^Full question: How much have your joint or muscle symptoms interfered with your work or daily routine in the last 2 weeks (including work and jobs around the house)? [44].

^b^Responses to health literacy question—extremely (likely high health literacy) to not at all (likely low health literacy).

^c^Self-efficacy measure [43] includes 6 items, scored from 1 (not at all confident) to 10 (totally confident).

#### Initial Visit

The 6 registered participants used the site for a mean duration of 21.6 minutes (SD 14.1) during their first visit (range 8.6-44.5). A total of 2 participants spent almost 3 minutes exploring the home page before moving on (likely viewing the GENIE information video), whereas the other participants moved on to register on the site almost immediately (6-13 seconds).

Overall, 2 participants, one with a diagnosis of OA and one without, viewed the “What is OA?” page first on the EMPOWER site, whereas 3 others chose to focus on issues that affected them (sleep, staying independent, and problem solving). The final participant opted to go to the goal setting page (*My goals*) first, discounting suggestions on the main menu to review information before setting goals. This participant (P20) chose not to visit any of the joint pain–related information pages during their initial visit, instead focusing on setting goals and visiting GENIE.

Data on the frequency of page views indicated that certain issues were more important than others. The *Being more active* and *Being a healthy weight and eating well* pages were the most visited, with one participant (P19) spending more time exploring this latter topic than any other. Information on medical management (accessed through *What is OA?*) was also of interest to 3 participants, with one user spending over 5 minutes exploring these 2 pages.

In total, 4 participants used the GENIE link button available in EMPOWER and used GENIE for 2 to ≥9 minutes before returning to EMPOWER. Multimedia Appendix 5 shows the process of going between the 2 sites. Participants did not use the option for recording “how could people around you help” and did not request a facilitated GENIE session. The EMPOWER site provided links to external sources of trusted information on the internet, but these were only used by one participant (Multimedia Appendix 5). Furthermore, links to videos from the Healthtalk website [57] providing real-life experiences of dealing with various issues were not used by participants.

#### Subsequent Visits

Overall, 2 participants returned to EMPOWER twice during the study, spending over 4 minutes (P15) and 19 minutes (P20) on the site and using GENIE. After logging in, these participants were given the opportunity to view their goals or go to the main menu. Both chose to view their goals initially, although only one of the participants had previously set a goal. This participant provided feedback on the progress of the previously set goals. Participants were invited to conduct evaluations of the intervention at the end of their month of use; however, these were not completed by any of the participants.

## Discussion

Guiding principles for the EMPOWER site and its integration with GENIE were established. These were associated with ensuring that EMPOWER was accessible, trustworthy, and relevant for different people with OA. Participants suggested that pain was the reason for seeking support but wanted a digital tool to go beyond medical information and provide opportunities to track progress. Think-aloud interviews using EMPOWER and GENIE highlighted user experience issues, particularly around linking sites and the personalization of information. Participants were divided over whether they would need to involve health care professionals with setting goals on EMPOWER. A greater focus on preventing pain in the future was identified as important, although achieving a relevant approach for all was considered difficult. The connection between EMPOWER and GENIE required greater explanation, both technically and conceptually. Adaptations and usability issues were resolved before the early evaluation work was undertaken.

Early evaluation work identified that activity and weight management were particularly relevant for participants. Most participants visited GENIE from the EMPOWER site on their first visit and follow-up visits. Goal setting and links to external information sources were only used by one participant, whereas video case studies were not used. Unfortunately, participants did not undertake the evaluations at the end of the study, which limited our understanding of the context of these decisions. The reasons for this are unclear, but it is possible that there were technical issues with the site, although this function was widely tested before going *live*. Digital evaluation (even with an email reminder) may have been too great an expectation, given the small number of additional visits to the site.

The domains of the NASSS framework (Multimedia Appendix 1) have been used to explore the intervention’s complexity and potential for implementation using the available usability data. These are discussed in the following sections.

### The Condition

OA can be considered complex, as many people do not have a diagnosis or are at different stages of managing the condition. OA was an unfamiliar term to some participants, which resulted in a change in the terminology used for recruitment. The EMPOWER home page was changed to accommodate this to meet the needs of those with lower levels of health literacy [59]. This approach was important, given that OA affects more people from lower socioeconomic backgrounds in which health literacy levels are also typically lower [6]. However, most of the recruited participants had high levels of health literacy. Although we aimed to recruit participants with diverse health literacy levels, we believe that our inability to do so may be symptomatic of the limited diversity of participants in health research more widely [60].

Pain was the primary reason for seeking support, and it was therefore important for participants to see the EMPOWER site before linking to GENIE. However, perceptions about the amount of information necessary to manage pain differed between participants. An association with aging may be one reason for this, as there are few perceived options for managing OA [61].

### The Technology

Technical issues associated with integrating EMPOWER and GENIE caused usability problems for participants, although adjustments were made to improve navigation between sites, with page flow data showing that participants were able to navigate from EMPOWER to GENIE and back again. The dependability of the sites was otherwise satisfactory, although it was apparent that some links did not always open on the initial click. It is unclear, however, whether this was a site issue or whether it was related to a user’s hardware or internet connection. Usability issues may reduce the effectiveness and potential of web-based interventions, and many self-management interventions are reported to have limitations in this area [62,63].

Although EMPOWER was developed to be independently usable, regardless of previous experience, the site emphasis was on goal setting to build efficacy for self-management behaviors. Goal setting is an effective strategy to support healthy behaviors, such as increased physical activity [64,65], which are recommended for the management of OA [12]. EMPOWER integrated the effective elements of goal setting and action planning, which are associated with the goal setting theory [51,52] and the health action process approach [51]. However, given that only one participant used this function in our evaluation, there may have been problems with the implementation of these functions or associated technical difficulties. Evaluating set goals is also a key part of goal setting [66], but few participants in this study returned to EMPOWER after the initial visit, potentially indicating ambivalence to this intervention. Finally, although there were frequent prompts to set goals, this may have assumed that individuals were able to develop, prioritize, and follow up on set goals. Given the participants’ range of self-efficacy scores (Table 3), this may have been too great an assumption.

We also made assumptions about the capability of participants to use the internet. Although a user guide was provided on how to navigate to the EMPOWER site, potential participants may have decided not to take part because of the self-directed nature of navigating to and using the site.

### The Value Proposition

The EMPOWER site was developed to support the self-management of joint pain for people with lower levels of health literacy. Previous research has identified that such interventions are also acceptable to those with higher levels of health literacy, with clear and accessible information preferred by individuals with both low and high health literacy [67-69]. The integration of EMPOWER and GENIE was considered valuable by encouraging users to move beyond a medicalized approach to self-management and think about what matters to them within their social practices and networks. Although this was theoretically informed and viable, there were technical issues and reported tensions related to mixed messages about self-efficacy. We suggest that this tension is caused by the underlying differences between the 2 sites. EMPOWER is a behavioral intervention, whereas GENIE is about social practices. The latter has been established to make a contribution that is not as immediate as needing to respond to and alleviate pain. Rather, it is designed to respond to the everyday demands of living life with a chronic condition [23]. In this context, GENIE supports people to seek activities that prevent or lessen the intensity of episodes over time (eg, through joining an activity group, which in turn may help to reduce episodes of acute pain).

EMPOWER was designed, based on user feedback, to be an initial point of contact for people seeking information and advice about joint pain. EMPOWER also promoted social engagement to support self-management by encouraging users to think about and record how their own social networks could help and to engage with GENIE. However, most participants did not engage with these features on the EMPOWER site. A facilitated approach has previously been found to encourage greater motivation and persistence to think about social network support using the GENIE site [23]. Such a facilitated approach was offered to participants using a simple electronic request on the EMPOWER site but was not taken up.

The tensions between the sites may have created practical and cognitive burdens for users, increasing the perceived complexity of the overall intervention. Although participants identified potential value in using a digital tool in collaboration with primary care, the increased complexity of the EMPOWER and GENIE intervention limits the potential for wide-scale future adoption [45].

### The Adopter System

Some of the focus group and interview participants suggested that health care professionals should introduce the sites’ resources and support goal setting. A lack of this input may have influenced the number of users registered during the evaluation phase. Previous studies have identified the importance of ensuring that new technologies contextually link with the existing health care environment [70]. In doing so, new interventions are more likely to be adopted and effective for their target population [71,72].

### Limitations

We aimed to recruit a diverse range of people to take part in developing a new self-management intervention for people with OA. Although we achieved our recruitment target for focus groups and interviews, a lack of diversity was evident with respect to health literacy. We further aimed to improve this during the evaluation study by focusing on recruitment areas with the highest indices of deprivation. However, most participants in this study were from areas with the lowest level of deprivation. Although study materials were designed to be user friendly and readable, this had little effect on recruiting people with lower levels of health literacy. We considered the library setting appropriate for recruitment as a provider of community information and support, particularly related to using the internet [73]. However, it may have been more appropriate to set up a local patient and public involvement group to better understand the needs of the local population first. This approach has been reported to be beneficial, alongside a snowball sampling strategy for engaging *hard-to-reach* groups in the community [74].

We were also unable to recruit many older adults (aged ≥75 years) to take part in the studies. Although people in this age category typically use the internet less than other age groups, data from the Office for National Statistics show increased use year-on-year [75], which we hope would translate into study participation. Consequently, the findings of this study cannot be generalizable.

During the focus groups and interviews, participants were asked to explore the websites in a way that was relevant to them. However, this may not have been representative of daily use, impacting on the validity of the results and subsequently the development and integration of the sites.

In the evaluation study, although the site was accessed 18 times during the data collection period, this only translated into 6 registered users. Although issues related to the technology and adopter system have been discussed as barriers to recruitment, some of the library locations were less engaged or were only open part-time, which may also have had an influence. Digital feedback forms were not completed by any participant, even with email reminders. It may have been prudent, therefore, to offer nondigital alternatives, such as postal questionnaires or telephone interviews, which may have been more acceptable. However, this would have required participants to provide their address and/or telephone number and may have influenced their decision to take part in the study.

Given the successes and limitations of this study, future work should incorporate close community partners working to understand the needs of the local population. In doing so, a more representative sample may be achieved, particularly in relation to levels of health literacy. Both health information and social network approaches are important for supporting self-management. However, future work should seek to clarify how best to integrate and implement these approaches in terms of digital or nondigital approaches and in the context of the wider health care system.

### Conclusions

This study has demonstrated the complexity associated with developing an accessible digital tool that combines self-efficacy and social network activation to support the variety of needs of people with joint pain. The NASSS framework provided a useful mechanism for evaluating and explaining the nonadoption of the intervention. Considerable complexity was associated with the integration of the EMPOWER and GENIE resources, both technically and conceptually. We identified tensions between the 2 resources caused by differences in their approach to self-management. Although EMPOWER aims to support change in self-management behaviors, GENIE focuses on building collective efficacy through engagement with valued activities. Recognition of joint pain as the prime reason for seeking support was considered important as was an association with health care professionals for some participants. However, connecting with the offline world was also considered beneficial through engagement with people and community resources for support. We therefore conclude that although an integrated approach was not adopted, both types of intervention have a role in the self-management of people with joint pain.

## Appendix

Multimedia Appendix 1 The 7 domains of the nonadoption, abandonment, scale-up, spread, and sustainability framework for the implementation of technology in health and social care, reproduced with permission and licensed under Creative Commons Attribution cc-by 4.0.

Multimedia Appendix 2 Focus group and think-aloud interview representative accounts.

Multimedia Appendix 3 Example Managing Joint Pain on the Web and Through Resources (EMPOWER) page during development showing the text input field for users to record how people around them could help.

Multimedia Appendix 4 Example of changes made following the think-aloud interviews.

Multimedia Appendix 5 Links between Managing Joint Pain on the Web and Through Resources (EMPOWER) pages and Generating Engagement in Network Involvement (GENIE).

## Abbreviations

EMPOWER: Managing joint Pain On the Web and through Resources
GENIE: Generating Engagement in Network Involvement
GP: general practitioner
LTC: long-term condition
MJP: My Joint Pain
NASSS: nonadoption, abandonment, scale-up, spread, and sustainability
NHS: National Health Service
OA: osteoarthritis
PBA: person-based approach
PIS: participant information sheet
