# Supplementary figures and images for: Integrating a Web-Based Self-Management Tool (Managing Joint Pain on the Web and Through Resources) for People With Osteoarthritis-Related Joint Pain With a Web-Based Social Network Support Tool (Generating Engagement in Network Involvement): Design, Development, and Early Evaluation
Source: JMIR Form Res. 2020 Nov 26;4(11):e18565. doi: 10.2196/18565 (PMC7728529; doi:10.2196/18565)

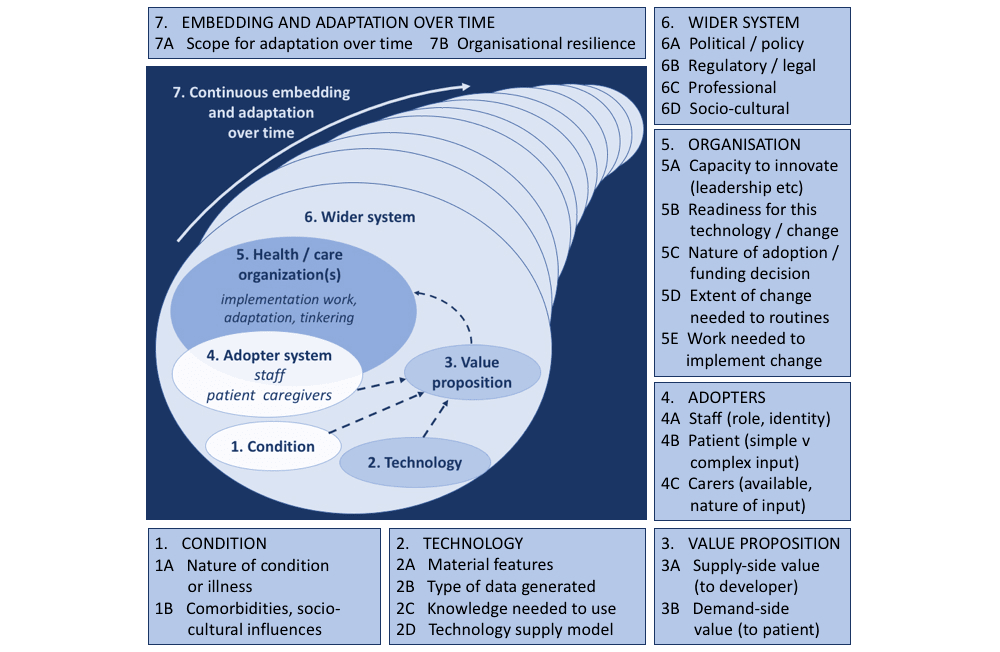

Supplement: Multimedia Appendix 1 [file formative_v4i11e18565_app1.png]

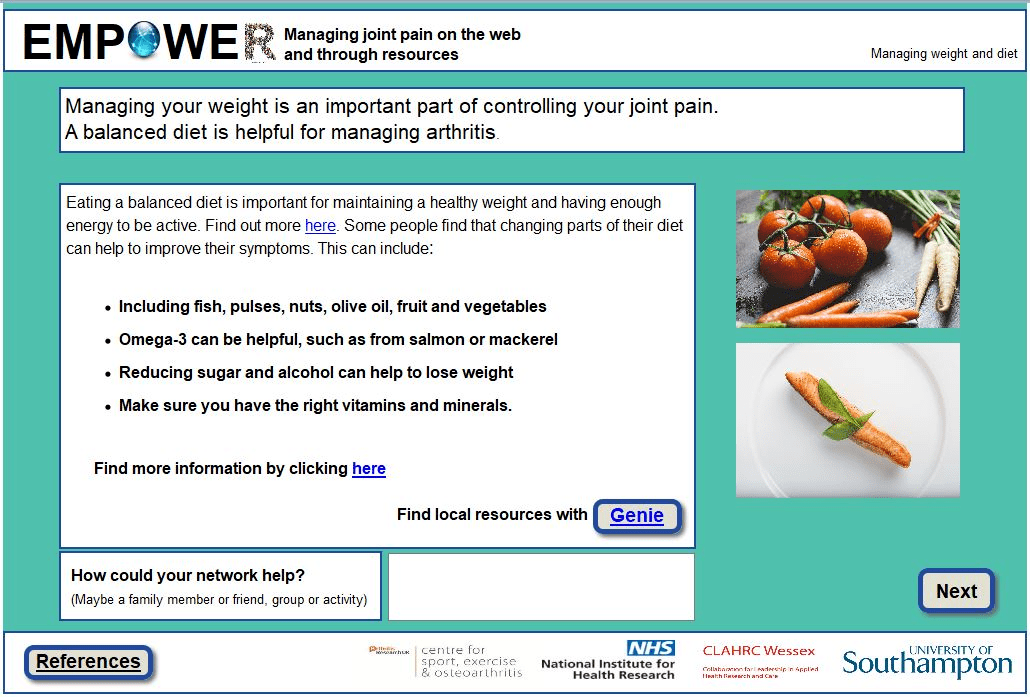

Supplement: Multimedia Appendix 3 [file formative_v4i11e18565_app3.png]

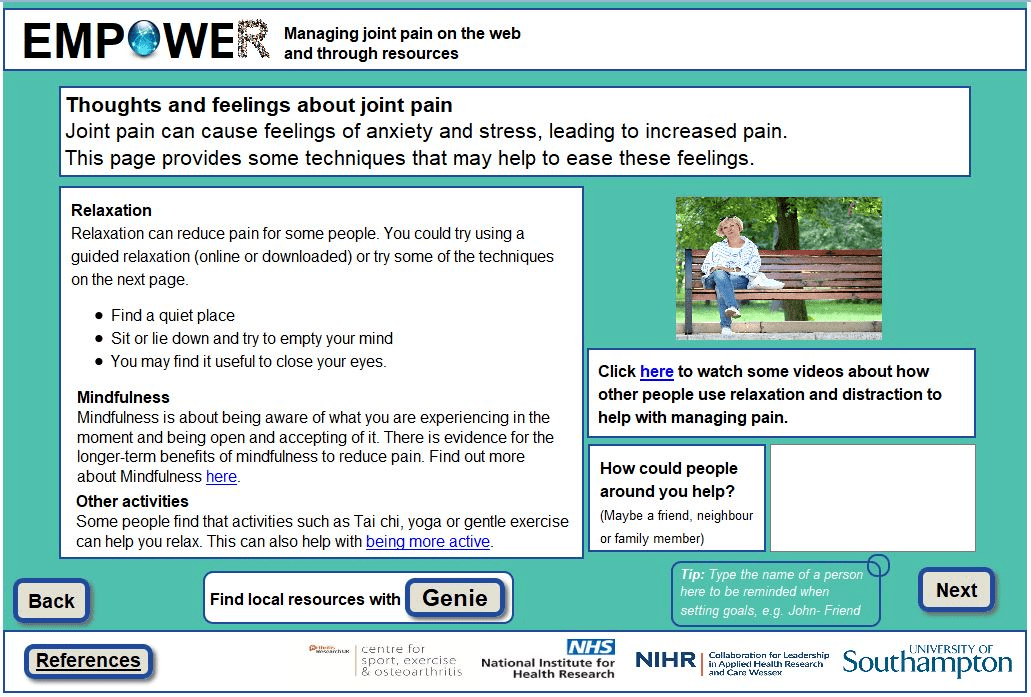

Supplement: Multimedia Appendix 4 [file formative_v4i11e18565_app4.png]

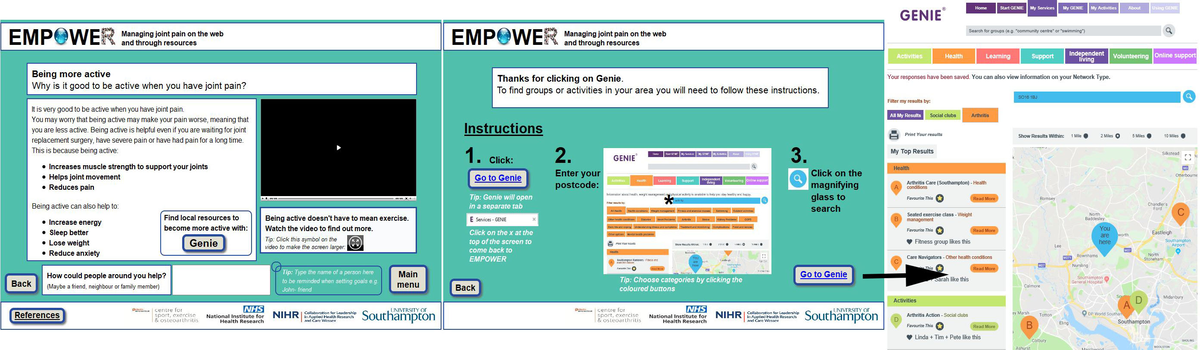

Supplement: Multimedia Appendix 5 [file formative_v4i11e18565_app5.png]
