# Supplementary material for: Integrating a Web-Based Self-Management Tool (Managing Joint Pain on the Web and Through Resources) for People With Osteoarthritis-Related Joint Pain With a Web-Based Social Network Support Tool (Generating Engagement in Network Involvement): Design, Development, and Early Evaluation
Source: JMIR Form Res. 2020 Nov 26;4(11):e18565. doi: 10.2196/18565 (PMC7728529; doi:10.2196/18565)
Supplement: Multimedia Appendix 2 [file formative_v4i11e18565_app2.docx]

Focus group participant quotes

| Flexibility | *“I also think the genie, you get part way through and you think, how’s this going to stop my thumbs from hurting or whatever, but [with] persistence you can see, if you have a narrow life how broadening, [could result in] bettering your life.” (P6)*  *“...I think if you’re going to do this [develop a connection between a joint pain SM site and Genie], you need the facility to switch rather than having to shut one down and open another one up...” (P3)* |
| --- | --- |
| Complementarity | *“The Genie in terms of getting you to do things, you know it’s all very well the first website [My Joint Pain] saying right be more active... I’ve found it better when they’ll [activity groups] do things in groups and that it’s not always easy [to] know what’s around and suitable.” (P7)*  *“I think it would be wonderful if they [the websites] could link in together, because I thought the first one was definitely factual and you would be able to think yes I’ve been given the absolute, the correct information...reliable, dependable info. Sometimes if you’ve got some sort of chronic condition and you’re in the wee small hours, you’re awake, you kind of need that social support where there might be on this Genie site...” (P14)* |
| Tailoring | *“I would prefer to have something that I could look at first and then have the option to sign up, you know like, sign up so that you can track your progress, I think that would be much more useful...” (P5)*  *“...I didn’t really know whether it was that personal in the end or whether they sort of tend to give the same prescription to everybody.” (P7)*  *“...I liked the pictures as well with the little dots [on the visual body map]… because I knew they’ve got a dot on the bit where I have the joint pain... it kind of reassured me in a way that …, I’m normal and that it happens to other people...” (P13)* |
| Important aspects of managing joint pain | *“There are a lot of young people who are caring, not necessarily full time and not necessarily acknowledged...who are clearly helping parents or grandparents cope with conditions…, so maybe there could be some sort of a thinking there.” (P14)*  *“...there’s not always an answer to things, its, you’ve got to learn sometimes to actually cope with it and live with it… and make the most of what you’ve got and of course the social side comes into that doesn’t it, yeah, so I think they’re both [sites] very helpful.” (P3)* |

Think aloud interview participant quotes

| User experience | “*I haven’t really been asked what my specific issue is, and I think that might be helpful at the very beginning...” (P13)*  *“See I don’t know things like that”* [referring to the use of separate browser windows for going between EMPOWER and Genie] *(P6)*  *“I ain’t very medically minded but it’s quite simple information there. It sort of gives you information that you want.” (P2)*  *“...I’m not sure I learnt a great deal personally” (P10)* |
| --- | --- |
| Professional involvement | *“...we can draw on our own experiences and our own personal knowledge already so it’s not about being in the hands of an expert” (P13)*  *“...on the video there saying about the physio can help you with this, and this area can advise you on that. But suddenly it’s just saying right just make up your mind what you think you want to do and do it” (P7)* |
| Tensions between EMPOWER and Genie | *“I’m wondering are these groups specifically set up all for people with problems, health problems? Or are they just anybody? They might not be very sympathetic if I’m walking along slowly at the back...” (P7)*  *“...I’m struggling [to see] how that would help me with my pain” [Referring to ‘Connecting with local people and activities’ page] (P10)* |
| Pain | *“I was expecting to see something more about how you lie in position if it’s your arm that aches try this or if your knee aches try this. I didn’t find anything and especially as this is to do with more pain related” (P10)*  *“I get trouble with my hands, but I don’t think it’s whatever that is” [referring to osteoarthritis] (P2)* |
| Goal setting | *“I like the idea of the fact of making me think about what I’ve read and now I can actually set myself some follow up tasks but my follow up tasks might be that I need to speak to someone about it first before I actually go ahead and do it in case I’m setting myself too big a step.” (P7)*  *“I’m struggling to know how I would set myself a goal here. If I was incredibly obese, how is writing this down going to actually help me achieve it.” (P10)* |
